# Religious subgroups influencing vaccination coverage in the Dutch Bible belt: an ecological study

**DOI:** 10.1186/1471-2458-11-102

**Published:** 2011-02-14

**Authors:** Wilhelmina LM Ruijs, Jeannine LA Hautvast, Koos van der Velden, Sjoerd de Vos, Hans Knippenberg, Marlies EJL Hulscher

**Affiliations:** 1 Academic Collaborative Centre AMPHI, Dpt of Primary and Community Care, Radboud University Nijmegen Medical Centre, Geert Grooteplein 21, 6525 EZ Nijmegen, The Netherlands; 2 Municipal Health Service GGD Rivierenland, Teisterbantlaan 1b, 4001 TJ Tiel, The Netherlands; 3 Dpt of Human Geography, Planning and Development Studies, University of Amsterdam, Nieuwe Prinsengracht 130, 1018 VZ Amsterdam, The Netherlands; 4 Scientific Institute for Quality of Healthcare, Radboud University Nijmegen Medical Centre, Geert Grooteplein 21, 6525EZ Nijmegen, The Netherlands

## Abstract

**Background:**

The Netherlands has experienced epidemics of vaccine preventable diseases largely confined to the Bible belt, an area where -among others- orthodox protestant groups are living. Lacking information on the vaccination coverage in this minority, and its various subgroups, control of vaccine preventable diseases is focused on the geographical area of the Bible belt. However, the adequacy of this strategy is questionable. This study assesses the influence of presence of various orthodox protestant subgroups (orthodox protestant denominations, OPDs) on municipal vaccination coverage in the Bible belt.

**Methods:**

We performed an ecological study at municipality level. Data on number of inhabitants, urbanization level, socio-economical status, immigration and vaccination coverage were obtained from national databases. As religion is not registered in the Netherlands, membership numbers of the OPDs had to be obtained from church year books and via church offices. For all municipalities in the Netherlands, the effect of presence or absence of OPDs on vaccination coverage was assessed by comparing mean vaccination coverage. For municipalities where OPDs were present, the effect of each of them (measured as membership ratio, the number of members proportional to total number of inhabitants) on vaccination coverage was assessed by bivariate correlation and multiple regression analysis in a model containing the determinants immigration, socio-economical status and urbanization as well.

**Results:**

Mean vaccination coverage (93.5% ± 4.7) in municipalities with OPDs (n = 135) was significantly lower (p < 0.001) than in 297 municipalities without OPDs (96.9% ± 2.1). Multiple regression analyses showed that in municipalities with OPDs 84% of the variance in vaccination coverage was explained by the presence of these OPDs. Immigration had a significant, but small explanatory effect as well. Membership ratios of all OPDs were negatively related to vaccination coverage; this relationship was strongest for two very conservative OPDs.

**Conclusion:**

As variance in municipal vaccination coverage in the Bible belt is largely explained by membership ratios of the various OPDs, control of vaccine preventable diseases should be focused on these specific risk groups. In current policy part of the orthodox protestant risk group is missed.

## Background

In the Netherlands the national vaccination program started in 1957. Despite a high vaccination coverage, in the last two decades there have been epidemics of poliomyelitis (1992-1993), measles (1999-2000), rubella (2004-2005) and mumps (2007-2008) [1-4]. These epidemics were all largely confined to an area stretching from the south-west to the north-east of the country, the so-called Bible belt, where -among others- orthodox protestant groups are living. Almost all patients in these epidemics belonged to the orthodox protestant minority and were unvaccinated because of religious objections.

Lacking information on the vaccination coverage in the orthodox protestant minority and its various subgroups, currently control of vaccine preventable diseases is focused on the geographical area of the Bible belt. Although the term Bible belt is generally understood as the area where the orthodox Protestants are living, the boundaries of this area are not exactly clear. It is often defined as municipalities with votes for the Staatkundig Gereformeerde Partij (SGP, the orthodox protestant political party) above a certain threshold, mostly 5% [5]. However, this percentage is set arbitrarily and the defined area is subject to change, e.g. because of municipal mergers of municipalities with higher and lower percentages of votes for SGP. So the adequacy of this policy to target a risk group for vaccine preventable diseases seems questionable. Knowledge of vaccination coverage in the orthodox protestant minority, and its various subgroups, could be helpful to focus prevention and control of vaccine preventable diseases at the persons really at risk.

The orthodox Protestants form a closed community within Dutch society [6]. They have their own churches, their own schools, their own newspaper and in politics they are represented by their own political party, the SGP. The orthodox protestant opposition to vaccination dates back to the 19^th ^century. At that time, like in other countries, severe side effects of smallpox vaccination fuelled in the Netherlands protests against compulsory vaccination [7,8]. Nowadays the main orthodox protestant arguments against vaccination focus on the necessity of trust in Divine providence, referring to certain passages in the Bible [9]. A different exegesis in favour of vaccination is, however, noticed as well among orthodox Protestants [10].

From the 19^th ^century on, a number of orthodox protestant denominations (OPDs) separated from the Netherlands Reformed Church. These OPDs not only vary in their interpretation of the Bible, they seem to vary in their position towards vaccination as well. In church periodicals from 1950's up to 2000 a tendency was observed from explicit rejection to stressing the personal responsibility and individual choice of church members. According to their periodicals the Reformed Congregations in the Netherlands and the Old Reformed Congregations seem to be most persistent in refusal [11].

Actual vaccination coverage among the various OPDs in the Netherlands is unknown. In the registration of the national vaccination program, religion is not recorded. Moreover, as religion is not recorded in any public registration, actual membership numbers of the OPDs are even largely unknown. Since vaccination is a sensitive subject among orthodox Protestants specific research on vaccination related issues in this minority is scarce and not differentiating among the various OPDs [12,13]. In the present study we will explore the influence of the various OPDs on municipal vaccination coverage in the Bible belt.

Apart from religious objections, the still remaining rural character of the Bible belt may influence vaccination coverage. Historically local churches of the OPDs were established in small villages in this area [14]. The presence of a large amount of orthodox Protestants in a small local community influences local culture. Church attendance among protestant groups, for example, appears to be more frequent if the relative size of the protestant group increases [15]. As social control interferes with personal choices that deviate from group norms, and social control is more prevalent in rural areas [16], the level of urbanization may be a determinant of municipal vaccination coverage in the Bible-belt.

In the Netherlands, preventive child care, including vaccinations conform the national vaccination programme, is offered free of charge to all children by child health clinics. The parents of all newborns are personally invited to visit these clinics, that are held in their neighbourhood. However, still not all eligible children may be reached. There might be cultural reasons for not attending the child health clinics. Internationally recent immigration and low socio-economical status are associated with low vaccination coverage [17-19]. These determinants may influence municipal vaccination coverage in the Bible-belt as well.

The aim of this ecological study is to explore the influence of the various OPDs on municipal vaccination coverage in the Bible belt. Knowledge of vaccination coverage in the orthodox protestant minority, and its various subgroups, could be helpful to focus prevention and control of vaccine preventable diseases at the persons really at risk.

## Methods

In order to achieve the aim of the study, the following research questions were formulated: Is there a difference in vaccination coverage between municipalities with and without OPDs? What is the influence of the membership ratios of separate OPDs (number of members of the OPD proportional to the total number of inhabitants of the municipality) on municipal vaccination coverage in municipalities where OPDs are present?

### Study design and population

An ecological study at municipality level was performed. All 458 municipalities in the Netherlands (reference date 01-01-2006) were included. As in the Netherlands municipal merging is an ongoing process and as in small municipalities churches may attract believers from neighbouring municipalities, in the provinces Zuid-Holland, Utrecht and Gelderland municipalities were aggregated for this study. In these provinces municipalities with less than 15.000 inhabitants were aggregated according to existing plans for municipal merger or according to geographical entities like (former) islands and polders. In this way 36 municipalities were aggregated to 10 geographical entities. Thus the study includes 432 municipalities and geographical entities, comprising all inhabitants of the Netherlands.

In this study the Bible belt is defined as all municipalities and geographical entities where one or more OPDs are established (irrespective of percentage of votes for SGP).

### Variables and data collection

#### Vaccination coverage

In this study vaccination coverage on municipal level was measured by the percentage of 2-year olds that completed DTPP (Diphteria Tetanus Pertussis Polio) vaccination according to scheme. To avoid fluctuations caused by small numbers of children in little villages the mean percentage was calculated for the years 2003, 2004 and 2005 (which were the most recent available data). The data on municipal vaccination coverage were obtained from the Health Inspectorate (2003) and from the RIVM, the National Institute for Public Health and the Environment (2004 and 2005).

#### Denomination

Membership numbers of all local branches of the five largest OPDs were gathered.

- Restored Reformed Church

The Restored Reformed Church does not publish membership numbers, therefore the local membership numbers were obtained from their central church office.

- Reformed Congregations

Local membership numbers of the Reformed Congregations were obtained from their Church Year Book.

- Reformed Congregations in the Netherlands

Local membership numbers of the Reformed Congregations in the Netherlands were obtained from their Church Year Book. For the in 1980 from the Reformed Congregations in the Netherlands seceded Reformed Congregations in the Netherlands (not synodally related) an estimate of the membership number was made based on literature [20]. In this small group a tendency is observed to return to their mother church, therefore these members were in this study added to the Reformed Congregations in the Netherlands.

- Old Reformed Congregations

The Old Reformed Congregations do not publish membership numbers, therefore the local membership numbers were obtained from their central church office. For the Free Old Reformed Congregations, who do not join the central church office, estimates of membership numbers were based on literature [20]. Because of religious kinship in this study the members of the free Old Reformed Congregations were added with the Old Reformed Congregations.

- Christian Reformed Churches

Local membership numbers of the Christian Reformed Churches were gathered from their Church Year Book. However, within the Christian Reformed Churches there are three different subgroups with an orthodox, intermediate or evangelical orientation. Therefore the orientation of each local branch was assessed by three informants belonging to this denomination. If at least two of them considered a local branch orthodox it was counted as such. Only the members of the orthodox branch were included in the analysis.

- Other orthodox protestant groups, not included in the study

Within the Protestant Church in the Netherlands (the largest Protestant denomination in the Netherlands) there are some members who sympathize with orthodox protestant exegesis. However as they are not registered as such we could not include them in our study. Another group we could not include is the small group of orthodox Protestants who do not join any denomination.

Subsequently, for every municipality in the Netherlands it was checked whether one or more local branches of the five above mentioned denominations were established in that municipality. And for those municipalities where one or more of these OPDs had been established, for each denomination the membership ratio was calculated by dividing the number of members of that specific OPD in the municipality by the total number of inhabitants of the municipality.

#### Urbanization

Classification of the urbanization of the municipalities was obtained from Statistics Netherlands. This classification is based on density of addresses and dichotomized in rural (<1000 addresses/km^2^) and urban (≥1000 addresses/km^2^).

#### Socio-economical status

Socio-economical status was indicated by the percentage of the population in a municipality that is receiving income support. Data were obtained from Statistics Netherlands, reference date 01-01-2006.

#### Immigration

Immigration was indicated by the percentage of non-western immigrants living in a municipality. Data were obtained from Statistics Netherlands, reference date 01-01-2006

#### Votes for SGP

The percentages of votes for SGP in the 2006 elections for parliament were obtained from Statistics Netherlands. Municipalities were dichotomized in municipalities with more and less than 5% votes for SGP.

The data obtained from Statistics Netherlands, RIVM and the Health Inspectorate are openly available via internet. The data on membership numbers of the OPDs were obtained via the churches, these data are not openly available.

### Analysis

Some variables had a somewhat skewed distribution, which leaves the use of parametric tests open to discussion. Therefore we performed non-parametric tests as well (N.B. both tests led to similar conclusions).

First, for all municipalities and geographical entities in the Netherlands, the effect of presence or absence of OPDs on vaccination coverage was assessed by comparing mean vaccination coverage using the independent samples t-test. As Levene's test for equality of variances was significant (p < 0.05) homogeneity of variance was not assumed, and therefore we used the corrected independent samples T-test in SPSS. Since the distribution of some variables was somewhat skewed we performed the Mann-Whitney test as well.

Second, for municipalities and geographical entities where one or more OPDs were present, the relationship between vaccination coverage and the explaining variables (membership ratios of the orthodox protestant denominations, urbanization, socio-economical status and immigration) was analysed by bivariate correlation (Pearson's r) and multiple regression analysis, using a backward selection method (removal criterion p > = 0.1). Since the distribution of some variables was somewhat skewed, Spearman's rho test was performed as well. Multiple regression analysis was repeated without the outliers responsible for skewed distribution of some variables. The residuals were all independent and normally distributed, there was no heteroscedacity and no collinearity.

Finally, to compare the influence of the membership ratios of various OPDs to the influence of over 5% votes for SGP the bivariate and multiple regression analyses were repeated with the variable >5% votes for SGP replacing the membership ratios of the OPDs.

## Results

### Characteristics of study population

Overall the OPDs in the Netherlands had almost 220,000 members. This means that 1.3% of the Dutch population is member of one of the OPDs. The membership numbers of the various OPDs on national level are shown in table 1.

**Table 1:** Orthodox protestant denominations on national level in the Netherlands

Denomination *(Dutch name of denomination)*	Datasource	Members	Living in municipalities with < 5% votes for SGP
Restored Reformed Church *(Hersteld Hervormde Kerk)*	Central Church Office	52690	6870 (13%)

Reformed Congregations *(Gereformeerde Gemeenten)*	Church Year Book 2006	103272	27258 (26%)

Reformed Congregations in the Netherlands* (Gereformeerde Gemeenten *in Nederland)*	Church Year Book 2007 Hoekstra 2008	24405	3483 (14%)

Old Reformed Congregations** (Oud Gereformeerde Gemeenten)	Central Church Office Hoekstra 2008	21192	5647 (27%)

Christian Reformed Churches*** (Christelijke Gereformeerde Kerken)	Church Year Book 2006 Personal communication	17547	6183 (35%)

Total		219106	49441 (23%)

*including Reformed Congregations in the Netherlands, not synodally related *(buiten verband)*

**including Free Old Reformed Congregations *(Vrije Oud Gereformeerde Gemeenten)*

***orthodox protestant subgroup, not including evangelical or intermediate subgroups

**Figure 1:**
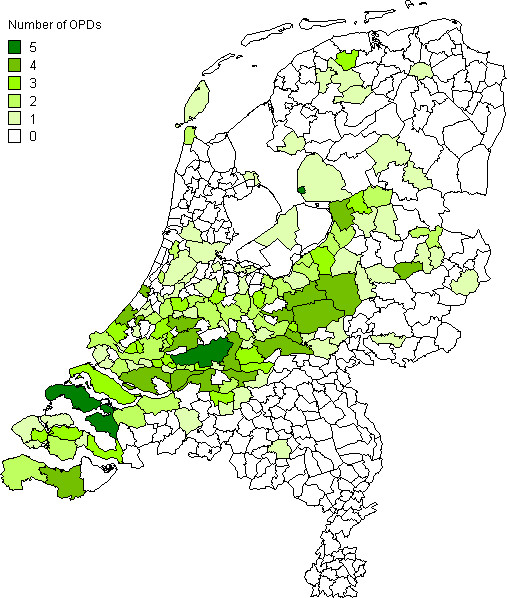
Number of OPDs per municipality or geographical entity

The 432 municipalities and geographical entities in our study had a mean population of 36,781 inhabitants. In 135 of these municipalities and geographical entities one or more OPDs were established (Table 2). Their geographical distribution is shown in Figure 1.

**Table 2:** Characterization of the municipalities and geographical entities, including vaccination coverage

Municipality or geographical entity	N	Mean % OPD members* (standard deviation)	Vaccination coverage (standard deviation)
Without OPD	297	-	96.9 (2.1)

With ≥ 1 OPD	135	4.9 (7.3)	93.5 (4.7)

1 OPD	60	1.4 (2.3)	96.0 (1.6)

2 OPDs	31	4.6 (7.1)	94.3 (3.5)

3 OPDs	22	8.7 (8.1)	91.9 (5.5)

4 OPDs	18	8.9 (6.7)	89.4 (5.1)

5 OPDs	4	20.6 (15.5)	82.4 (8.6)

* % OPD members = total number of members of all OPDs in the municipality combined, proportional to the population of the municipality

In only 41 (30%) of these 135 municipalities and geographical entities the percentage votes for SGP was over 5%. Almost a quarter of the orthodox Protestants is living outside municipalities and geographical entities with more than 5% votes for SGP.

### Vaccination coverage in relation to presence of OPDs

Including all municipalities and geographical entities, mean vaccination coverage was 95.8% (SD 3.5). In the 297 municipalities without OPDs mean vaccination coverage was 96.9% (SD 2.1) whereas in the 135 municipalities and geographical entities where one or more OPDs were established mean vaccination coverage was 93.5% (SD 4.7). The mean vaccination coverage of municipalities with at least one OPD (93.5%) is statistically significant lower than the mean vaccination of municipalities without OPDs (96.9%) (P < 0.001). As the number of OPDs established in a municipality or geographical entity increases, mean vaccination coverage decreases (Table 2).

### Influence of individual OPDs on vaccination coverage

In municipalities and geographical entities where one or more OPDs were established we assessed the influence of the individual OPDs on vaccination coverage, as well as the influence of urbanization, immigration and socio-economical status.

In table 3 for the 135 municipalities and geographical entities with OPDs, the bivariate correlations, using Pearson's r, between the vaccination coverage and the independent variables are shown. As expected, membership ratios of all OPDs have negative correlations with vaccination coverage, meaning that higher membership ratios are related with lower vaccination coverage. Level of urbanization showed the expected positive relation: meaning that in urban areas the vaccination coverage was higher than in rural areas. At first sight the proportions of non-western immigrants and of people dependent on income support (indicating socio-economical status) showed unexpected positive relations with vaccination coverage. This can be explained, however, because non-western immigrants and people dependent on income support mainly live in urbanized areas where the OPDs are underrepresented. Repeating bivariate correlation using Spearman's rho gave comparable results, except for the Christian Reformed Churches (rho = - 0.17, p = 0.053) and for level of urbanization (rho = 0.13, p = 0.131)

**Table 3:** Influence of OPD membership ratios on municipal vaccination coverage

	N = 135 municipalities or geographical entities with OPDs					N = 128 municipalities or geographical entities with OPDs, leaving out 7 outliers				
**Explaining variables**	**Pearson**		**multiple** **regression**			**Pearson**		**multiple** **regression**		

	r	p	b	t	p	r	p	b	t	p

Constant			97.15	313.20	<0.001			97.00	296.91	<0.001

% Restored Reformed Church*	-0.45	<0.001	-0.37	-7.32	<0.001	-0.46	<0.001	-0.43	-4.97	<0.001

% Reformed Congregations*	-0.63	<0.001	-0.49	-10.85	<0.001	-0.65	<0.001	-0.41	-6.78	<0.001

% Reformed Congregations in the Netherlands*	-0.66	<0.001	-1.57	-9.17	<0.001	-0.61	<0.001	-1.70	-6.27	<0.001

% Old Reformed Congregations*	-0.65	<0.001	-1.22	-6.83	<0.001	-0.61	<0.001	-1.37	-5.86	<0.001

% Christian Reformed Churches*	-0.38	<0.001	-0.36	-4.00	<0.001	-0.14	ns#			

% Nonwestern immigrants**	0.23	<0.001	-0.07	-2.54	<0.05	0.17	<0.05	-0.06	-2.22	<0.05

% With income support**	0.22	<0.05				0.18	<0.05			

Level of urbanization***	0.28	<0.001				0.24	<0.001			

Explained variance			85%					73%		

Results of bivariate correlation and multiple regression analysis, showing influence of OPD membership ratios, immigration, socio-economical status and urbanization on vaccination coverage (in municipalities and geographical entities with OPDs, and leaving out outliers)

* % OPD = membership ratio of OPD = percentage of municipal population that is member of the named orthodox protestant denomination

** % = percentage of municipal population consisting of non western immigrants respectively percentage of municipal population with income support

*** dichotomized variable: rural = 0, urban = 1

# ns = not significant

Table 3 also shows the result of a multiple regression analysis, using a backward selection method (removal criterion p > = 0.1). Level of urbanization and socio-economical status did not have any explanatory effect. A percentage of 84 of the variance in vaccination coverage was explained by membership of the various OPDs. The b-values all showed the expected negative sign but varied for the various denominations. The largest denominations -the Reformed Congregations and Restored Reformed Church- both had b-values around -0.40. This implies that 1 per cent point increase in membership ratio is associated with only 0.40 per cent point decrease in vaccination coverage. For the Reformed Congregations in the Netherlands and the Old Reformed Congregations, b-values exceeded minus 1, which implies that 1 per cent point increase in membership ratio is associated with even more than 1 per cent point decrease in vaccination coverage. Immigration had a significant, but very small explanatory effect; the total explanation only increased to 85%. The b-value now showed the expected negative sign.

Seven municipalities or geographical entities, all strongholds of certain OPDs, were recognized as outliers. Compared to the other municipalities and geographical entities they had an extremely high membership ratio for one OPD, which might have had an undue influence on the analysis. Repeating the analysis in 128 municipalities or geographical entities, leaving out the strongholds, 73% of variance in vaccination coverage could be explained. Again level of urbanization and socio-economical status had no significant effect, and here the membership ratio of Christian Reformed Churches was removed as well. All other results are comparable with the analyses on the basis of 135 geographical entities (Table 3).

In order to check if indeed the various OPDs were influential or merely the total membership ratio of all OPDs in the municipality, we repeated the analyses replacing the membership ratios of the various OPDs by the total membership ratio of all OPDs together. However, in this way the explanation of variance in vaccination coverage was 77% (versus 85%) in the group of 135 municipalities and geographical entities, and 64% (versus 73%) in the group of 128.

### Municipalities with more and less than 5% votes for SGP

As the Bible belt is often defined as municipalities with more than 5% votes for SGP, we repeated the bivariate correlation and multiple regression analysis replacing the membership ratios of the OPDs by the dichotomized variable more than 5% votes for SGP. In bivariate correlation more than 5% votes for SGP was, as expected, negatively correlated to vaccination coverage. Multiple regression analyses showed a negative b-value as well, however the explanation of variance in vaccination coverage was only 45% in the group of 135 geographical entities, and 43% in the group of 128, (Table 4).

**Table 4:** Influence of votes for SGP on municipal vaccination coverage

	N = 135 municipalities or geographical entities with OPDs					N = 128 municipalities or geographical entities with OPDs, leaving out 7 outliers				
**Explaining variables**	**Pearson**		**multiple** **regression**			**Pearson**		**multiple** **regression**		

	r	p	b	t	p	r	p	b	t	p

Constant			96.25	212.55	<0.001			95.81	336.14	<0.001

Votes for SGP*	-0.66	<0.001	-6.40	-9.42	<0.001	-0.66	<0.001	-5.24	-9.75	<0.001

% Non western immigrants**	0.23	<0.001				0.17	<0.05			

% With income support**	0.22	<0.05				0.18	<0.05			

Level of urbanization***	0.28	<0.001	1.54	1.69	0.094	0.24	<0.001			

Explained variance			45%					43%		

Results of bivariate correlation and multiple regression analysis, showing influence of votes for SGP, immigration, socio-economical status and urbanization on vaccination coverage (in municipalities and geographical entities with OPDs, and leaving out outliers)

* dichotomized variable: < 5% votes for SGP = 0, >5% votes for SGP = 1

** % = percentage of municipal population consisting of non western immigrants respectively percentage of municipal population with income support

*** dichotomized variable: rural = 0, urban = 1

To assess the influence of the membership ratios of the various OPDs on vaccination coverage in municipalities with less than 5% votes for SGP, we repeated the bivariate correlation and multiple regression analyses in these municipalities and geographical entities where OPDs were present (n = 94). In that case 26% of variance in vaccination coverage could be explained, the membership ratios of the two most conservative OPDs (the Reformed Congregations in the Netherlands and the Old Reformed Congregations) and the percentage non-western immigrants had a significant influence. The b-values for both conservative OPDs exceeded again minus 1.

## Discussion

Municipalities and geographical entities with OPDs had significantly lower vaccination coverage than municipalities without OPDs. Variance in vaccination coverage in municipalities and geographical entities with OPDs could largely be explained by the membership ratios of the various OPDs. This suggests that membership of an OPD is an important factor in explaining individual vaccination choice. However, we did not have data on the individual level and all relations were established on the level of municipalities, so in order to avoid the ecological fallacy, translation to individual relations has to be done with care.

### Vaccination coverage among orthodox Protestants

In multiple regression the largest denominations - the Reformed Congregations and Restored Reformed Church - both had b-values around -0.40. Although our analysis was not at an individual level, this finding is a strong indication that a substantial part of the members of these denominations is vaccinated.

Two smaller denominations - the Reformed Congregations in the Netherlands and the Old Reformed Congregations - had the strongest negative relation with vaccination coverage. This is in line with the negative statements on vaccination in their church periodicals [11]. In multiple regression analysis for both denominations the b-value exceeds -1. This could be explained assuming that the members of these two denominations not only reject vaccination for themselves but that they influence others (from their own and other denominations) to reject vaccination as well. Another possible explanation for the b-values exceeding minus 1, however, is the age distribution within these two denominations. In the Netherlands as well as in the United States orthodox protestants refrain from family planning, their families are large and the young members of the denomination outnumber the older members [6,21]. As in our study vaccination coverage is assessed at two years age and membership proportion is according to the total population, the proportion members of these denominations in the two years age cohort might exceed the proportion in the total population. Nevertheless, the findings strongly suggest that vaccination coverage in these denominations is very low.

The orthodox protestant subgroup of the Christian Reformed Churches is the smallest denomination in our study. The b-value in the multiple regression analysis suggests that a substantial part of the members are vaccinated. In our second analysis, leaving out among others two strongholds of the Christian Reformed Churches, their influence on vaccination coverage was not significant anymore.

As in current policy the Bible-belt is often defined as municipalities with more than 5% votes for SGP, we replaced the membership ratios of the OPDs by the variable more than 5% votes for SGP. However, in this way the explanatory effect regarding variance in vaccination coverage is considerably lower.

### Limitations of the study

The present study has some limitations. In this ecological study analyses could only take place on the municipality level, which hinders drawing conclusions on the individual level. Moreover municipalities were included regardless of their population number, and multiple regression analyses were not weighted for municipal population size. This means that small and large municipalities have an equal impact on the results. The aim of our study is, however, to assess the relationship between the membership ratios of the various OPDs (measured as percentage) and the municipal vaccination coverage (measured as percentage as well). The relationship itself (measured as b-value) is not dependent on the size of the municipality and we corrected for possible confounding factors such as urbanization, immigration and low socio-economical standard. Thus multiple regression analyses weighted for municipal population size would not affect the conclusions regarding the b-values of the various OPDs.

Misclassification may occur if members of OPDs do not live in the municipality where their church is seated. We partly corrected for this problem by aggregation of small and medium sized municipalities to geographical entities like (former) islands and polders. However, municipalities where an OPD is seated might have been allocated more members of that OPD than really live in this municipality. Vaccination coverage is always measured according to home address. Since the negative correlations between OPD and vaccination coverage probably would have been even more pronounced when both variables would have been measured according to home address, this inconsistency does not interfere with our conclusions.

Finally, variance in vaccination coverage could not be completely explained by the variables in our model. In international literature low vaccination coverage is related to lack of health insurance, lack of reimbursement and lack of long term preventive care [17,22,23]. In the Netherlands this is not expected to be a problem as vaccinations according to the NVP are provided free of charge. Travelling communities, like Roma and Irish travellers are associated with outbreaks of vaccine preventable diseases [24]. However, in the Netherlands travelling communities are not expected to influence municipal vaccination coverage as these travelling communities are small (an estimated 20,000 persons) and vaccination coverage among children travelling with fairs was comparable to the general Dutch population[25]. Finally, apart from religious objections to vaccination, parents may have philosophical objections or a critical attitude towards vaccination because of perceived side effects [26,27]. These non-religious objections are often confined to MMR-vaccination as measles, mumps and rubella are considered to be useful diseases to strengthen the immune system [28]. As these objections are not registered we could not take them into account. However, we tried to minimize their influence by choosing DTPP-vaccination coverage as the dependent variable. Orthodox protestant objections concern all vaccinations.

Until now preparedness for epidemics of vaccine preventable diseases in the Netherlands has been focused on the geographical area of the Bible belt, often defined as municipalities with over 5% votes for SGP. However, 23% of the OPD-members is living outside this area and our study showed that in municipalities with less than 5% votes for SGP, where OPDs are present, the membership ratios of the most conservative OPDs still have a significant influence on vaccination coverage. In current policy, orthodox Protestants living outside the area defined as Bible belt are not addressed by health promotion and vaccination campaigns during epidemics. The orthodox Protestants constitute a closed community maintaining almost all social contacts within their own group [6] and orthodox Protestants living outside the municipalities with more than 5% votes for SGP are at considerable risk for infection during epidemics. Therefore we suggest to include them in prevention and control measures. As the orthodox Protestants have a strong social infrastructure, public health workers may seek cooperation with orthodox protestant intermediaries like schools or patients' associations, in order to prevent and control outbreaks of vaccine preventable diseases.

## Conclusion

Municipal vaccination coverage in the Dutch Bible belt is largely dependent on the membership ratios of the various OPDs. Control of vaccine preventable diseases should therefore be focused on these religious risk groups.

## Competing interests

The authors declare that they have no competing interests.

## Authors' contributions

WLMR conceived of the study, participated in the design, collected the data, participated in statistical analyses and drafted the manuscript. JLAH participated in the design of the study and helped to draft the manuscript. KvdV participated in the design of the study and revised the manuscript. SdV participated in statistical analyses and the interpretation of the data and revised the manuscript. HK participated in the design of the study and interpretation of the data and revised the manuscript. MEJL participated in the design of the study and helped to draft the manuscript. All authors read and approved the final manuscript.

## Authors' information

WLMR is preparing a thesis on "Acceptance of vaccination in orthodox protestant groups". This is the first exploratory study of this thesis. Further research is focusing on vaccination coverage within the various orthodox protestant denominations and individual decisions of members of these denominations whether or not to vaccinate one's children.

## Pre-publication history

The pre-publication history for this paper can be accessed here:

http://www.biomedcentral.com/1471-2458/11/102/prepub
